# Reviewed article: Reis RS, Schincaglia RM, Moreira MM, Ferreira JS, Gouveia PF, Mortoza AS, Guimarães MM, Peixoto MRG, Souza LB. Association between commensality practices and healthy food consumption in Primary Care: a cross-sectional study, Goiânia, 2022-2023. Epidemiol Serv Saude. 2025:34;e20240367

**DOI:** 10.1590/S2237-96222025v34e20240367.a

**Published:** 2025-10-27

**Authors:** Nathália Luíza Ferreira

**Affiliations:** 1 Universidade Federal de Lavras, Lavras, MG, Brazil Universidade Federal de Lavras Lavras MG Brazil

## Peer review A

## Questionnaire

Does the manuscript contain new and significant information to justify publication? Yes

Does the Abstract (Summary) clearly and accurately describe the content of the article? No

Is the problem significant and concisely stated? No

Are the methods described comprehensively? Yes

Are the interpretations and conclusions justified by the results? Yes

Is adequate reference made to other work in the field? No

## Manuscript Structure

Length of article is: Adequate

Number of tables is: Adequate

Number of figures is: Adequate

Please state any conflict(s) of interest that you have in relation to the review of this paper. None

Interest: Excellent

Quality: Good

Originality: Good

Overall: Good

Recommendation: Major Revision

## Comments

Trabalho com temática relevante. No entanto, há questões a aprimorar, com destaque para a necessidade de maior clareza quanto ao objeto de estudo e à sua justificativa. Ademais, as análises focam na investigação de fatores associados às variáveis denominadas pelos autores como de exposição, e não às variáveis consideradas como desfechos. Assim, a condução das análises precisa melhor dialogar com a proposta.

O título está extenso. Alguns termos poderiam ser suprimidos.

Resumo

Em um primeiro contato com o trabalho, as variáveis não ficam tão claras. Deixar claro que são marcadores do SISVAN.

As práticas, poderiam ser denominadas como práticas promotoras da comensalidade, por exemplo.

Ex. Adesão a práticas promotoras da comensalidade e alimentação saudável.

Assim, fica mais claro o objeto do estudo.

Os escores foram criados pelos autores? A qualidade dos escores foi testada?

Como a amostra foi obtida? Por que consiste em estudo de base populacional? Não ficou claro no resumo.

Seria interessante no resumo ter as prevalências das práticas e do consumo. Pelo menos os mais prevalentes, por serem as variáveis centrais do estudo.

Importante a conclusão se limitar à população estudada e aos dados analisados.

Introdução

p. 3, linha 3: “relação entre as práticas alimentares motivadas pelo Guia e a qualidade da dieta dos brasileiros, uma vez que essa aumenta o risco para diversas doenças crônicas não transmissíveis, de relevância epidemiológica”

Esse trecho dá a entender que a qualidade da dieta acarreta doenças. Parece contraditório. Sugiro reestruturar a sentença.

Métodos

A recusa de uma das unidades básicas de saúde implicou em substituição por outra unidade?

Há dados mais recentes sobre IDH-M.

Página 4. Por que não adotar os procedimentos antropométricos preconizados pelo SISVAN?

Página 4. Linha 53. Foram utilizados dados referidos? Não ficou claro.

Página 5. O número amostral calculado não foi atingido. Qual o impacto deste fato? Qual a justificativa?

Página 5. Seria interessante explicar a motivação para adoção do modelo de regressão em questão.

Resultados

Da forma que os dados no texto e na configuração das tabelas, parece que os desfechos são as práticas alimentares. Nos Métodos, são indicados como desfechos os marcadores de consumo. Importante rever qual a proposta de análise e deixar o texto compatível com o que foi avaliado.

Os fatores associados são investigados em relação às práticas, e não ao consumo, por exemplo. Há um descompasso nesse sentido.

Discussão

Os autores poderiam aprofundar mais na Discussão.

Poderia ser melhor explorada a comparação com a literatura. Os estudos que avaliaram a relação investigada com a amostra do Nutrinet, por exemplo, obtiveram resultados semelhantes?

Como a maior perda de homens pode impactar os resultados?

De modo geral, os autores, ao longo de todo o texto, inclusive no título, poderiam focar

mais na comensalidade para justificar a investigação do consumo alimentar.

